# Blood Pressure Effects and Risk of Hypotension due to Intravenous Furosemide in Acute Decompensated Heart Failure

**DOI:** 10.1111/acem.70125

**Published:** 2025-08-28

**Authors:** Nicholas E. Harrison, Meghana Bhaskara, Kyle Wilson, Ankit A. Desai, Nicholas Montelauro, Phillip Levy, Peter Pang, Robert R. Ehrman

**Affiliations:** ^1^ Department of Emergency Medicine Indiana University School of Medicine Indianapolis Indiana USA; ^2^ Division of Cardiology Indiana University School of Medicine Indianapolis Indiana USA; ^3^ Department of Emergency Medicine Wayne State University School of Medicine Detroit Michigan USA

## Abstract

**Objective:**

We quantified the magnitude of systolic blood pressure (SBP) adverse effects associated with intravenous furosemide (IVFu), compared to other factors, during treatment for acute decompensated heart failure (ADHF).

**Methods:**

Continuous BP monitoring (598.2 person‐hours, 91,210 observations) before and after IVFu was performed in a prospective multicenter ADHF cohort (*n* = 253). Multivariable‐adjusted mixed effects regression was used to determine the amount of SBP reduction and the risk of hypotension attributable to IVFu administration, as opposed to confounders (e.g., non‐IVFu treatments and baseline patient characteristics).

**Results:**

Median SBP was 124 mmHg (IQR: 105–149) at baseline. Hypotension occurred in 5515 observations (6.0%). The multivariable models explained 79.6% and 58.1% of variance in SBP and risk of hypotension, respectively. Only 1.4% of variance in SBP and 1.7% of hypotension risk were related to IVFu, with the remainder accounted for by confounders. After multivariable adjustment, SBP dropped −11.9 mmHg on average after 80 mg IVFu, reaching a nadir at 147 min (−15.2 mmHg) and partial return to baseline by 6 h (−8.5 mmHg). IVFu‐related risk of hypotension after multivariable adjustment depended predominantly on baseline SBP and dose. Risk of hypotension associated with 80 mg IVFu was ≤ 2% with baseline SBP ≥ 120 mmHg. For 40 mg, IVFu‐associated hypotensive risk was ≤ 2% with SBPs of 90–100 mmHg, and < 1% with SBP ≥ 110 mmHg. IVFu‐associated risk of hypotension returned to zero at 6 h after administration, regardless of dose.

**Conclusions:**

Blood pressure reductions after IVFu during ADHF treatment are modest, and hypotension is rare and transient. Most variance in SBP during ADHF treatment is due to other factors.

## 1 Introduction

One million Americans are treated every year for acute decompensated heart failure (ADHF), and intravenous (IV) loop diuretics are the backbone of guideline‐recommended medical management [1, 2]. Hypotension is an adverse effect of IV furosemide ubiquitously referred to in popular clinical references (e.g., “UpToDate,” “Micromedex,” “AccessMedicine”) [3, 4, 5]. Interestingly, we are aware of no literature evaluating IV furosemide‐related effects on blood pressure in an ADHF population. While furosemide‐related hypotension is described in healthy volunteers and patients with chronic compensated heart failure, data are lacking on the rate and predictors of hypotension in ADHF patients.

Understanding the effects of IV furosemide on systolic blood pressure (SBP) during ADHF treatment is critical, because the fear of hypotension may lead clinicians to withhold this necessary therapy or administer it at lower doses than guidelines recommend [1, 2, 6]. Up to 50% of ADHF patients are discharged from the hospital without receiving enough IV loop diuresis to achieve complete decongestion, which is in turn associated with a three‐ to sixfold increase in short‐term ADHF‐related readmission or death [1, 7, 8].

Additional data on IV loop diuretic‐related risk of hypotension in the ADHF population specifically will help clinicians make informed decisions about harm avoidance during standard of care decongestive therapy. Our objective was to characterize trends in SBP and rates of hypotension before versus after IV loop diuretic treatment in ADHF, analyzing continuous blood pressure monitoring adjusted for confounders and time‐varying effects.

## 2 Methods

### 2.1 Study Design and Setting

This multicenter prospective observational study was approved as minimal‐risk research by the Wayne State University (WSU) and Indiana University (IU) institutional review boards. This cohort study prospectively enrolled patients from five hospital emergency departments (EDs) in two US states. A detailed description of the study design, from which this pre‐planned secondary analysis was performed, has been published previously as CLEAR‐AHF [9]. This manuscript was prepared according to the STROBE guidelines [10].

### 2.2 Selection of Participants

Patients with ED physician suspicion of ADHF and any of the following were included: dyspnea due primarily to ADHF per physician judgment, pulmonary edema on chest radiograph (CXR), and/or BNP (N‐terminal pro B‐type natriuretic peptide) > 300 pg/mL. Exclusion criteria were temperature > 38.5°C, suspected sepsis, ST‐elevation myocardial infarction, pregnancy, prisoners, unknown ejection fraction (EF) within 12 months, and patients without diagnostically adjudicated ADHF.

### 2.3 Interventions

After enrollment, two study authors with extensive ADHF research experience (P.P. and P.L.), blinded to one another, reviewed cases to adjudicate the ED diagnosis of ADHF. Disagreements were decided by discussion. A ClearSight technician, blinded to the analysis and clinical data, reviewed all monitor waveforms offline and excluded measurements where device internal diagnostics suggested a low‐quality signal (e.g., if a patient adjusted or removed the monitor to get up to the bathroom).

Clinical decision‐making by the treating physician determined the interventions (e.g., IV furosemide, noninvasive positive pressure ventilation [NIPPV], and nitroglycerin) used for ADHF. Interventions were recorded based on the ClearSight monitor's internal clock to harmonize the timing of clinical and hemodynamic data. No other IV loop diuretics were used in the hospital besides IV furosemide.

### 2.4 Measurements

#### 2.4.1 Demographics and Treatment Data Collection

Research assistants used standardized data sheets to record patient demographics, medications, medical history, vital signs, clinical tests, and ED treatments. Data were obtained through the electronic medical record (EMR), and the reports of treating physicians were recorded in REDCap (Research Electronic Data Capture; http://project‐redcap.org/).

#### 2.4.2 Blood Pressure Monitoring

Patients with ADHF meeting inclusion and exclusion criteria were fitted with a ClearSight finger‐cuff monitor (Edwards Lifesciences, Irvine, California). The device continuously monitors BP and heart rate (HR) at the digital and radial arteries to noninvasively reconstruct an arterial pressure waveform. Measurements of systolic, diastolic, and mean arterial pressures (SBP, DBP, MAP) and HR were timestamped to 20‐s intervals for regression analysis. A prior version of the device (“NexFin”) was validated for *R* ^2^ = 0.96 agreement with invasive blood pressure monitoring [11]. A recent meta‐analysis found an average difference of 4.2 mmHg (95% CI: 2.8–5.6) between finger‐cuff monitor BP and invasive gold standard (i.e., arterial line). Patients were monitored continuously for 3–6 h.

### 2.5 Outcomes

The following clinical data were collected prospectively: SBP, DBP, MAP, HR, interventions, and timing and dose of IV loop diuretics. The primary study outcome was SBP. The primary comparison was the difference in SBP relative to administration, timing, and dose of IV loop diuresis, as opposed to other interventions and patient factors. The dose of furosemide used was the dose deemed clinically appropriate by the treating emergency department physician. SBP was analyzed as a continuous variable and as a binary for hypotension, defined as SBP < 90 mmHg. A secondary outcome of “sustained hypotension” was defined as mean SBP < 90 mmHg across 10‐, 20‐, and 30‐min periods.

### 2.6 Statistical Analysis

Mixed effects regression modeling was employed to account for within‐subject and between‐subject variation, and to perform multivariable adjustment for confounders of the association between IV loop diuresis and SBP. Two models were fit, a linear mixed model (LMM) of SBP as a continuous variable and a generalized LMM (GLMM) logistic regression for presence/absence of hypotension at any time point (*n* = 91,210). The primary fixed‐effects predictor of interest was administration of IV loop diuresis, including interaction terms for minutes since administration (modeled with a restricted cubic spline to allow for time‐varying effects), diuretic dose (in IV furosemide equivalents, FEq), and home diuretic status (chronic loop diuretic therapy versus diuretic‐naïve). Based on two important pharmacokinetic considerations, we chose to model outcomes up to 6 h after IV loop diuretic administration (and any amount of time beforehand). First, the time to peak effects and terminal half‐life of IV furosemide are approximately 2 h, but duration of action can extend as long as 6 [12]. Second, the “braking‐phenomenon” of IV loop diuretics has an onset around 6 h, and therefore, repeat administrations are typically given 6–8 h after an initial dose [12].

Use of IV nitroglycerin, NIPPV, and cumulative furosemide dose were fit as random intercepts to adjust for suspected clustering on blood pressure; a random intercept for unique patients modeled within‐subject correlation. Total monitoring time was fit as a random slope. Expected confounders (fixed effects in each model for multivariable adjustment) included: baseline vital signs (SBP, DBP, respiratory rate, oxygen saturation), total time monitored, ED therapies besides IV loop diuretics, age, sex, body mass index (BMI), HR (repeated measurement), medical history (hypertension, diabetes, chronic heart failure, myocardial infarction, renal disease, atrial arrhythmias, valvular heart disease, COPD), pre‐ED home medications, ED labs (troponin, BNP, sodium, potassium, blood urea nitrogen {BUN}), EF, and presence/absence of atrial fibrillation/flutter on the ED electrocardiogram (ECG). Interaction terms included baseline SBP versus each ED therapy (IV or PO renin‐angiotensin‐aldosterone system blockers {ACEi, ARB}, IV or PO beta‐blockers, IV or PO calcium channel blockers {CCB}, PO loop diuretics, IV or sublingual nitroglycerin, NIPPV, supplemental oxygen). Interactions between these therapies and IV loop diuretic administration were further included to test for effects of co‐administrations. Variable distributions of the response and random effects were evaluated to ensure distributional model assumptions were met. Relationships to the outcomes were assessed, and continuous variables were transformed to maximize linearity where appropriate. Model residuals were evaluated for homoscedasticity.

The LMM and GLMMs were tested for fit by Nagelkerke's adjusted *R* ^2^ statistic, representing the proportion of variance in each outcome explained by the model [13, 14, 15]. *R* ^2^ values were calculated with and without the IV loop diuretic model terms to determine how much variance in SBP and hypotension was explained by IV loop diuresis versus other multivariable‐adjusted factors. Adjusted mean difference by parameters of interest (e.g., SBP with versus without IV calcium channel blocker) was evaluated by model effects estimated at the median values or majority class (i.e., with exception of the variable of interest). Patients with missing data (*n* = 4) were excluded. Comparisons of baseline variables were calculated (alpha = 0.05) using Wilcoxon rank sum (continuous) and Chi Squared or Fisher's exact test (categorical). Analyses were performed in R.

## 3 Results

### 3.1 Characteristics of Study Subjects

Of 330 patients enrolled, 257 were adjudicated as ADHF, and 4 were excluded due to missing data. The analysis included 91,210 observations (598.2 person‐hours of monitoring) from 253 patients. A detailed inclusion and exclusion diagram has been published previously [9]. A majority of patients (85% vs. 92% with/without IV loop diuresis, Table 1) had ≤ 10 cumulative minutes of hypotension. A total of 6% of observations (*n* = 5150) were hypotensive (< 90 mmHg).

**TABLE 1:** Patient and treatment characteristics.

Characteristic	Overall, *N* = 253^a^	No IV loop diuretic, *n* = 76^a^	IV loop diuretic, *n* = 177^a^	*p* ^b^
*Baseline characteristics and home medications*				
Age	60 (50, 67)	60 (48, 68)	60 (51, 67)	0.7
Female sex	114 (45%)	36 (47%)	78 (44%)	0.6
BMI	32 (26, 39)	30 (26, 36)	33 (26, 40)	0.053
Systolic blood pressure, triage	154 (132, 178)	156 (139, 183)	153 (129, 175)	0.2
Diastolic blood pressure, triage	91 (79, 105)	93 (80, 103)	91 (78, 106)	0.8
Heart rate, triage	92 (81, 105)	93 (80, 105)	92 (81, 105)	> 0.9
Respiratory rate, triage	20 (18, 22)	20 (18, 23)	20.0 (18.0, 22.0)	0.2
Oxygen saturation, triage	97 (95, 99)	97 (95, 98)	98 (95, 99)	0.3
Hypertension	236 (93%)	69 (91%)	167 (94%)	0.3
Diabetes	125 (49%)	35 (46%)	90 (51%)	0.5
Dialysis	19 (7.5%)	10 (13%)	9 (5.1%)	0.026
Pulmonary hypertension	74 (29%)	20 (26%)	54 (31%)	0.5
Atrial fibrillation or flutter	48 (19%)	11 (14%)	37 (21%)	0.2
Myocardial infarction	80 (32%)	21 (28%)	59 (33%)	0.4
Heart failure	234 (92%)	69 (91%)	165 (93%)	0.5
Valvular heart disease	90 (36%)	27 (36%)	63 (36%)	> 0.9
COPD	97 (38%)	25 (33%)	72 (41%)	0.2
Ejection fraction	40 (25, 59)	45 (25, 58)	40 (23, 60)	0.5
ACE inhibitor	91 (36%)	25 (33%)	66 (37%)	0.5
Angiotensin receptor blocker (ARB)	32 (13%)	14 (18%)	18 (10%)	0.070
Beta blocker	176 (70%)	56 (74%)	120 (68%)	0.4
Calcium channel blocker	75 (30%)	27 (36%)	48 (27%)	0.2
Home loop diuretic	154 (61%)	36 (47%)	118 (67%)	0.004
Nitroglycerin	58 (23%)	15 (20%)	43 (24%)	0.4
Troponin I	0.03 (0.00, 0.07)	0.03 (0.00, 0.06)	0.03 (0.00, 0.08)	0.9
Brain natriuretic peptide	1070 (414, 2055)	1007 (422, 2344)	1107 (414, 2024)	0.7
Sodium	139.0 (137.0, 142.0)	138.0 (136.0, 140.0)	140.0 (137.0, 142.0)	< 0.001
Potassium	4.10 (3.80, 4.50)	4.10 (3.78, 4.50)	4.10 (3.80, 4.50)	> 0.9
BUN	21 (15, 31)	21 (13, 33)	21 (16, 30)	0.3
Atrial fibrillation on ED ECG	21 (8.3%)	6 (7.9%)	15 (8.5%)	0.9
*Treatment characteristics*				
Intravenous nitroglycerin	28 (11%)	11 (14%)	17 (9.6%)	0.3
Noninvasive positive pressure ventilation	16 (6.3%)	5 (6.6%)	11 (6.2%)	> 0.9
Supplemental oxygen	65 (26%)	19 (25%)	46 (26%)	0.9
ACE inhibitor or ARB	29 (11%)	6 (7.9%)	23 (13%)	0.2
Beta blocker	69 (27%)	23 (30%)	46 (26%)	0.5
IV diltiazem	4 (1.6%)	0 (0%)	4 (2.3%)	0.6
Oral dihydropyridine CCB	21 (8.3%)	7 (9.2%)	14 (7.9%)	
Oral diltiazem	1 (0.4%)	0 (0%)	1 (0.6%)	
No CCB	227 (90%)	69 (91%)	158 (89%)	
Oral loop diuretic	6 (2.4%)	6 (7.9%)	0 (0%)	< 0.001
Intravenous loop diuretic dose			40 (40, 40)	NA
Initial systolic blood pressure at IV loop diuretic administration			148 (126, 174)	NA
*Events*				
Total minutes of hypotension				
0	98 (39%)	32 (42%)	66 (37%)	0.6
1–10	123 (49%)	38 (50%)	85 (48%)	
11–20	8 (3.2%)	1 (1.3%)	7 (4.0%)	
21–30	4 (1.6%)	0 (0%)	4 (2.3%)	
31–60	12 (4.7%)	2 (2.6%)	10 (5.6%)	
61–120	7 (2.8%)	3 (3.9%)	4 (2.3%)	
> 120	1 (0.4%)	0 (0%)	1 (0.6%)	
Proportion of time hypotensive	0.4% (0.0, 3.1)	0.3% (0.0, 2.6)	0.6% (0.0, 3.2)	0.3
Sustained hypotension ≥ 10 min	11 (4.3%)	4 (5.3%)	7 (4.0%)	0.7
Sustained hypotension ≥ 20 min	10 (4.0%)	4 (5.3%)	6 (3.4%)	0.5
Sustained hypotension ≥ 30 min	0 (0%)	0 (0%)	0 (0%)	NA

^*a*^ Median (IQR); *n* (%).

^*b*^ Wilcoxon rank sum test; Pearson's Chi‐squared test; Fisher's exact test.

Patients had a median (interquartile range [IQR]) age of 60 (50–67), EF 40% (25%–59%), and BNP 1070 (414‐2055). Further baseline and treatment characteristics, presented in Table 1, differed between patients receiving versus not receiving IV loop diuretics with regard to dialysis history, home loop diuretic use, serum sodium, and ED administration of oral loop diuretics (*p* < 0.05). Other characteristics were similar with versus without diuresis (Table 1). The median IV loop diuretic dose administered was 40 mg FEq, and the median SBP was 148 mmHg (126–174 mmHg) at the time of administration.

### 3.2 Systolic Blood Pressure and Hypotension Before vs. After Intravenous Loop Diuretics

**FIGURE 1:**
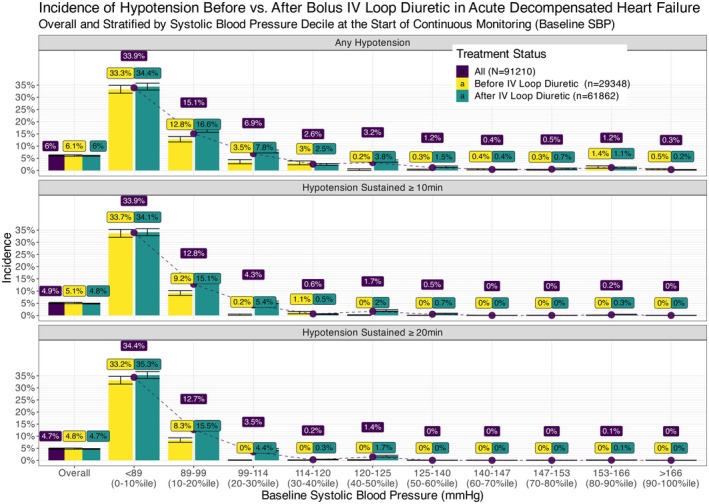
Incidence of hypotension stratified before versus after intravenous loop diuresis and by decile of baseline systolic blood pressure (SBP). Continuous measurements, *n* = 91,210, were taken every 20 s from 253 patients with acute decompensated heart failure.

On average, SBP decreased 6 mmHg lower after IV loop diuretic administration (*p* < 0.001, Table 2). Incidence of hypotension was 6.0% across observations overall, with similar rates before versus after IV loop diuretics (6.1% vs. 6.0%, *p* = 0.7). While 155 patients had at least one measured SBP < 90 mmHg, the vast majority were non‐sustained. The rates of hypotension sustained for ≥ 10 and ≥ 20 min were 4.9% and 4.7%, respectively, with similar rates before/after diuresis (Table 2). No episodes of hypotension were sustained for ≥ 30 min. Incidence of hypotension, including sustained hypotension, was most common with baseline SBP < 100 mmHg. With increasing baseline deciles of SBP, the incidence of hypotension decreased (Figure 1). No patients required vasopressors or other blood pressure support.

**TABLE 2:** Blood pressure before vs. after intravenous loop diuretic.

Characteristic	Overall, *N* = 91,210^a^	Before IV loop diuretic, *n* = 29,348^a^, ^b^	After IV loop diuretic, *n* = 61,862^a^	*p* ^c^
Systolic blood pressure, mmHg	136 (115, 158)	140 (118, 163)	134 (114, 156)	< 0.001
Diastolic blood pressure, mmHg	79 (67, 91)	79 (66, 91)	79 (68, 91)	0.6
Mean arterial pressure, mmHg	99 (85, 115)	101 (85, 118)	99 (85, 114)	< 0.001
Heart rate, bpm	84 (73, 95)	83 (71, 94)	85 (74, 96)	< 0.001
Hypotension (Systolic blood pressure < 90 mmHg)	5515 (6.0%)	1794 (6.1%)	3721 (6.0%)	0.7
Sustained hypotension ≥ 10 min	4483 (4.9%)	1485 (5.1%)	2998 (4.8%)	0.2
Sustained hypotension ≥ 20 min	4320 (4.7%)	1415 (4.8%)	2905 (4.7%)	0.4
Sustained hypotension ≥ 30 min	0 (0%)	0 (0%)	0 (0%)	NA

^a^ Median (IQR); *n* (%).

^b^ Includes observations of patients who did not receive a diuretic during the monitoring period.

^c^ Wilcoxon rank sum test; Pearson's Chi‐squared test.

### 3.3 Multivariable‐Adjusted Effect of IV Loop Diuresis on Systolic Blood Pressure

**FIGURE 2:**
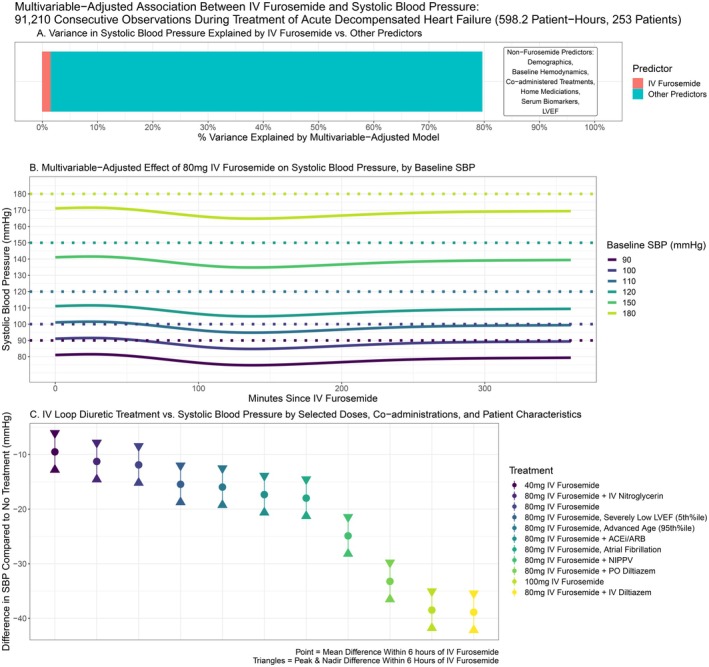
Multivariable‐adjusted association between IV furosemide and systolic blood pressure (SBP). (A) Variance in SBP during acute decompensated heart failure (ADHF) treatment explained by IV furosemide versus other factors. (B) Multivariable‐adjusted effect of 80 mg IV furosemide on SBP, stratified by baseline (pre‐treatment) blood pressure. (C) Differences in multivariable‐adjusted association of IV furosemide and SBP by dose, patient characteristics, and co‐administered treatments.

After multivariable adjustment, IV loop diuresis (including dose in FEq and minutes since administration up to 6 h) explained only 1.4% of the overall variance in SBP (Figure 2A). Overall, the multivariable model explained 79.6% of SBP variation during the 598.2 h of ADHF patient monitoring (Figure 2A, *n* = 91,210 observations).

Figure 2B presents the multivariable‐adjusted trends in SBP over the 6‐h period following an 80 mg IV furosemide dose, stratified by “baseline” SBP at the time of administration. The shape of the trend included an early nadir in SBP around 147 min (Figure 2B), consistent with furosemide's 2‐h peak effect. SBP thereafter increased, followed by a plateau, without completely returning to baseline by 6 h (Figure 2B). Comparing 80 mg of IV furosemide to no treatment at the median baseline SBP of 145 mmHg, the multivariable‐adjusted mean, nadir, and peak post‐diuresis differences in SBP were −11.9, −15.2, and −8.5 mmHg, respectively. Doses of 40 mg and 100 mg were associated with lesser versus greater changes in SBP, respectively (Figure 2C).

The 78.1% of SBP variation not attributed to IV furosemide was accounted for by 36 observed confounders, along with co‐administered treatments having multiplicative effects on SBP when IV furosemide was given (i.e., significant model treatment interactions). Patient‐level characteristics with multivariable‐adjusted associations with lower SBP were lower EF, advanced age, lower baseline SBP, higher BMI, diabetes, outpatient beta blocker use, higher troponin, hyponatremia, hypokalemia, bradycardia on the continuous monitor, and atrial fibrillation on ED ECG (*p* < 0.05). Higher SBP was associated with histories of hypertension and/or chronic HF, outpatient use of ACE inhibitors, ARBs, or nitroglycerin, and higher BNP.

The effects of selected co‐administrations and patient characteristics after receiving 80 mg IV furosemide are presented in Figure 2C. Many co‐administrations with IV furosemide had multiplicative effects on SBP. Co‐administration of furosemide with ACE inhibitors or ARBs (adjusted mean difference {MD} treatment vs. no treatment −17.3 mmHg), NIPPV (MD: −24.9 mmHg), and oral diltiazem (−33.2 mmHg) was associated with a significantly greater reduction in SBP than furosemide and the co‐administered treatment alone (p interaction < 0.05). Nitroglycerin and IV furosemide co‐administration was associated with a similar SBP reduction as furosemide alone when baseline SBP was normal‐high, but a significant multiplicative effect at the lowest deciles of baseline SBP. Diltiazem, both with (MD: −46.9 mmHg IV, −32.3 mmHg PO) and without furosemide (−32.8 mmHg IV, −21.83 mmHg PO), was the strongest treatment predictor of lower SBP.

### 3.4 Multivariable‐Adjusted Risk of Hypotension After IV Loop Diuresis

**FIGURE 3:**
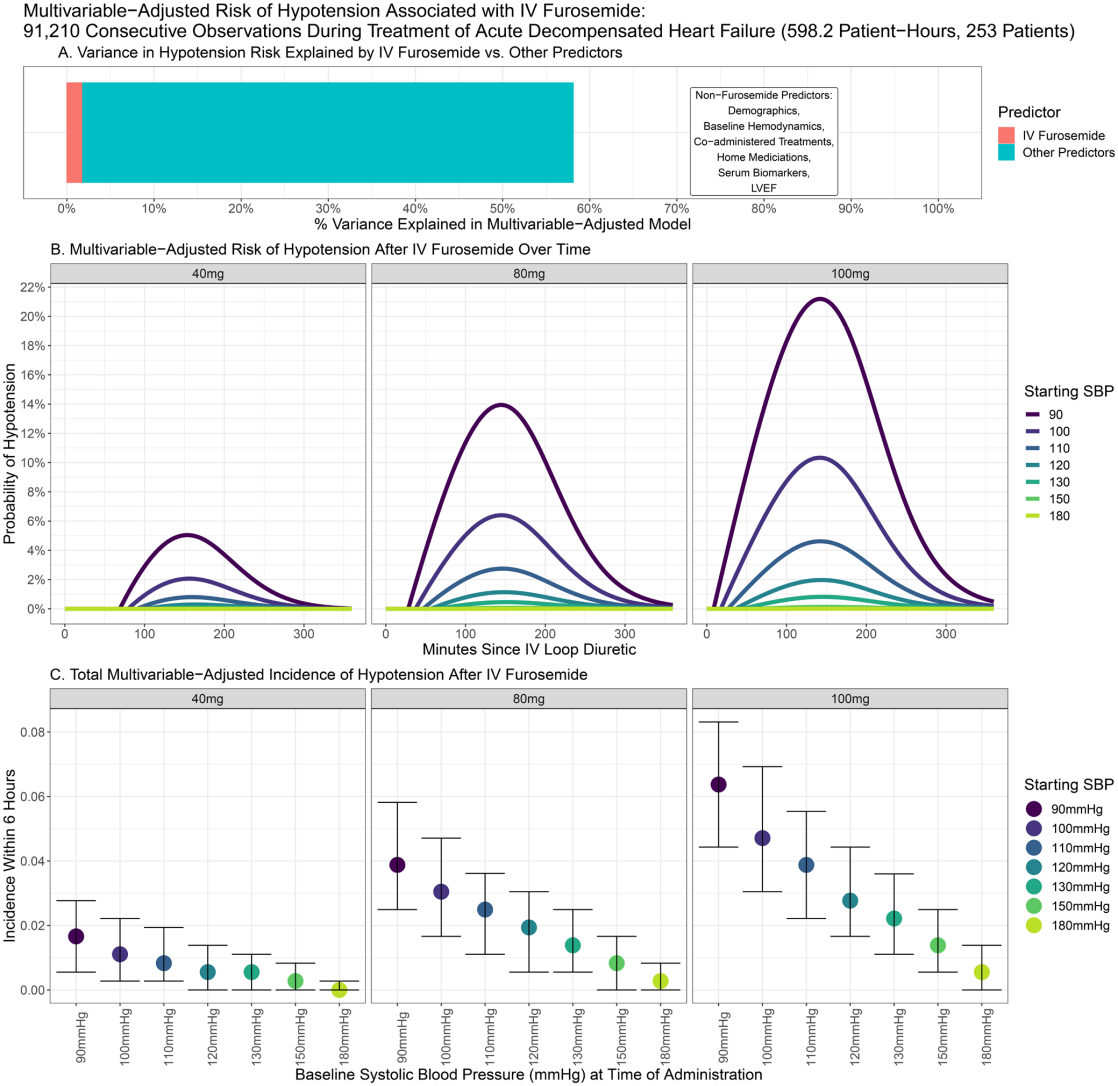
Multivariable‐adjusted risk of systolic hypotension associated with IV furosemide. (A) Variance in hypotension risk during acute decompensated heart failure (ADHF) treatment explained by IV furosemide versus other factors. (B) Multivariable‐adjusted risk of hypotension associated with IV furosemide over time, up to 6 h following administration, and stratified by baseline (pre‐treatment) blood pressure and dose (40, 80, or 100 mg). (C) Cumulative multivariable adjusted incidence of hypotension associated with IV furosemide during the 6 h after administration, stratified by baseline blood pressure and IV furosemide dose. Error bars = 95% confidence.

The second multivariable model explained 58.1% of the overall variance in risk of hypotension, with 1.7% related to IV furosemide and 56.4% related to non‐furosemide confounders (Figure 3A). Figure 3B presents the multivariable‐adjusted risk of hypotension associated with IV furosemide over time, stratified by dose and baseline SBP. Like the SBP model, the risk of hypotension increased with higher doses of furosemide and lower baseline SBP and varied over time with peaks between 100–200 min (Figure 3B).

Total incidence of hypotension related to IV furosemide during the 6 h following administration is presented in Figure 3C. For all doses of furosemide, the risk of hypotension was dramatically lower at baseline SBPs ≥ 110 mmHg compared to 90–100 mmHg. Furosemide‐related incidence of hypotension overall was < 1%, < 3%, and < 4% for 40 mg, 80 mg, and 100 mg (respectively) at baseline SBPs of 110–180 mmHg. At SBP baselines of 90–100 mmHg, the incidence of 1%–2%, 3%–4%, and 4%–7% respectively were estimated for the same doses (Figure 3C).

Other significant multivariable‐adjusted predictors of hypotension besides IV furosemide included lower baseline SBP, male sex, outpatient beta blocker use, uremia, and ED treatment with IV diltiazem (*p* < 0.05). IV nitroglycerin was associated with significantly less hypotension at normal‐high baseline SBP and greater hypotension at low baselines. Outpatient ACE inhibitor use, low HR on continuous monitoring, and ED treatment with PO or IV beta blockers and NIPPV were associated with lower rates of hypotension. Co‐administration of NIPPV or beta blockers with IV furosemide, however, was associated with increased hypotension risk.

## 4 Discussion

**FIGURE 4:**
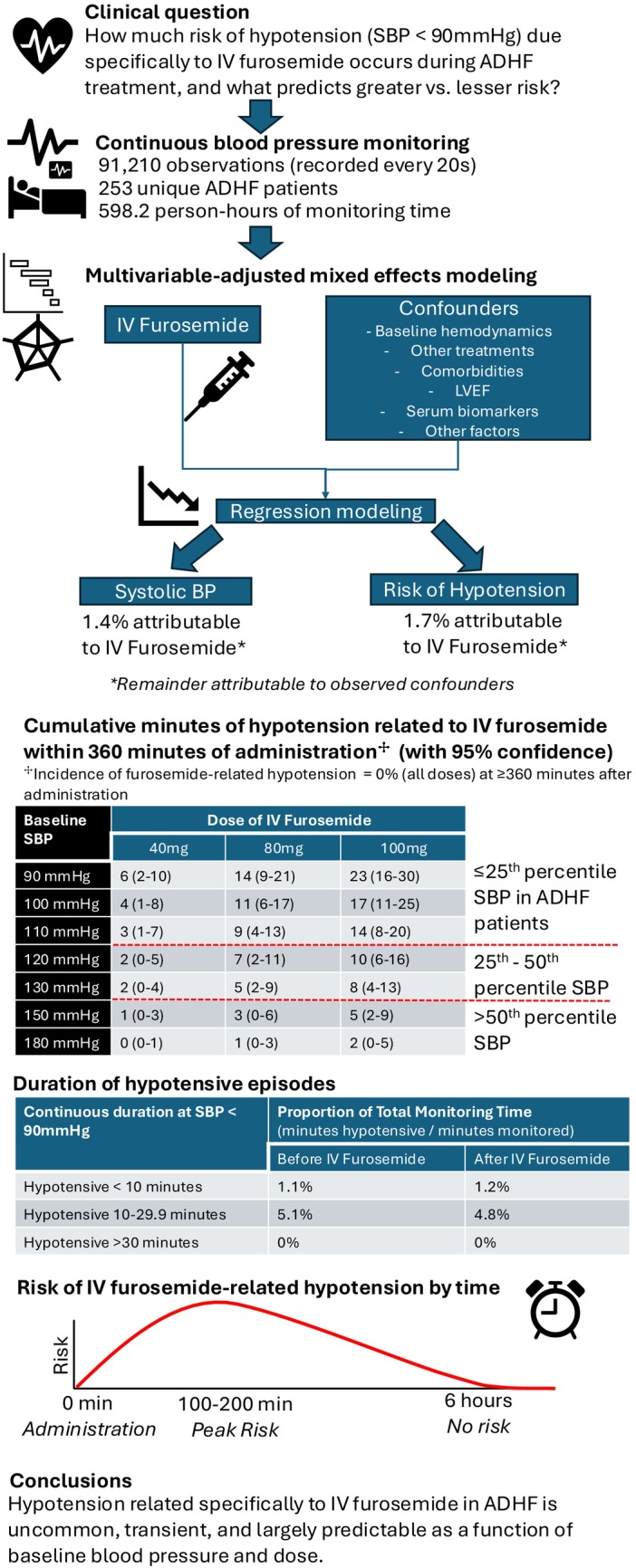
Central Illustration and Key Study Findings. Continuous blood pressure monitoring, recorded every 20 s (*n* = 91,210 observations, 598.2 person‐hours), was performed in a multicenter prospective ADHF cohort (*n* = 253 patients). Episodes of hypotension (SBP < 90 mmHg) were recorded and analyzed before versus after IV furosemide. Multivariable‐adjusted mixed effects regression modeling was used to identify the contribution of IV furosemide versus confounders to the incidence of hypotension and identify modifiers that increased the risk of furosemide‐related hypotension over time. ADHF, acute decompensated heart failure; IV, intravenous; min, minutes; SBP, systolic blood pressure.

We describe, for the first time, the blood‐pressure effects and risk of hypotension associated with IV furosemide in ADHF patients. Our data should be reassuring, especially finding that the association between SBP and IV furosemide within 6 h of administration is modest at commonly used doses (Figure 2B), and all but 1.4% of variance in SBP over time was explained by factors besides IV furosemide (Figure 2A). Hypotension was similarly rare when administered to patients with baseline SBP above 110 mmHg (Figure 4). Risk of hypotension associated with 80 mg IVFu was ≤ 2% with baseline SBP ≥ 120 mmHg. For 40 mg, IVFu‐associated hypotensive risk was ≤ 2% with SBPs of 90–100 mmHg, and < 1% with SBP ≥ 110 mmHg.

These rates of furosemide‐associated hypotension arguably appear even less clinically significant when one considers a few additional factors. First, there was no observed risk of hypotension due to IV furosemide after 6 h (i.e., all hypotension associated with IV furosemide was transient, regardless of dose or starting SBP). Second, zero patients experienced hypotension sustained for ≥ 30 consecutive minutes (Table 2), also regardless of dose or baseline SBP (Figure 3B). Third, the cumulative minutes of furosemide‐related hypotension across the 6‐h monitoring period were few. With a baseline SBP of 120 mmHg, administration of 40 mg of IV furosemide was associated with an average of 2 cumulative minutes of hypotension (95% CI: 0–5 min) during 6 h of post‐diuretic monitoring (Figure 4). The same baseline blood pressure yielded 7 total minutes (2–11) of hypotension after 80 mg IV furosemide, and 10 min (6–16) after 100 mg.

Lower blood pressure and higher dose were the primary correlates of hypotension risk related specifically to IV furosemide. The 100 mg dose was associated with a nearly 4‐fold decrease in SBP compared to 80 mg (−38.4 mmHg, Figure 2C), and higher rates of hypotension. Still, the maximum cumulative duration of hypotension related to IV furosemide, which occurred at starting SBPs of 90 mmHg and doses of 100 mg, was just 23 min (16–30 min), or 6.3% of the 6‐h monitoring time (Figure 4). In a typical ED patient with blood pressure monitoring occurring every 30–60 min (as opposed to every 20 s in our study), such a small duration of hypotension may go unnoticed during typical clinical blood pressure checks. Moreover, few ADHF patients will ever have a baseline SBP as low as 90 mmHg, making this “maximal” 23‐min cumulative duration of hypotension largely inapplicable to most scenarios. In large multinational registries, the mean SBP at hospitalization for ADHF patients has been reported at around 130–140 mmHg [16, 17]. Given the transience of hypotension risk observed and most patients presenting with elevated baseline SBP, the benefit of aggressive diuresis will therefore outweigh hypotension risk in many cases where a high dose is clinically indicated (e.g., patients on high PO doses and signs of loop diuretic resistance [2, 18]). Overall, our findings should help clinicians recognize that baseline SBP may increase the risk of hypotension, allowing more patient‐specific treatment choices.

Patient‐level factors, such as severely low EF, advanced age, and atrial fibrillation, significantly increased blood pressure reduction and risk of hypotension in the ED. In patients with these features, it may be reasonable to administer lower doses of furosemide (e.g., 40 mg) when pre‐treatment SBP is borderline to low (e.g., < 110 mmHg). By examining the confounding effects of co‐administered treatments with and without IV furosemide, we found that multiple co‐administered treatments also had profound effects on SBP which, at times, were multiplicative with IV furosemide's effects. Some of these are likely unavoidable based on specific clinical situations, such as NIPPV in patients with respiratory failure.

Others, like calcium channel blockers, are relatively contraindicated in heart failure per AHA guidelines [1]. Use of diltiazem in combination with furosemide was the single greatest multivariable‐adjusted predictor of reduced SBP (Figure 2C), which underscores that these agents should be avoided or used with extreme caution in ADHF patients who require IV loop diuresis. Patients administered ACEi/ARB alongside IV furosemide had small but significantly greater SBP reduction than those with equivalent IV furosemide doses alone. Other medications administered in the ED (e.g., beta blockers, MRAs, dihydropyridine CCBs) did not show significant effects on SBP reduction; but the analysis was not specifically powered to detect subtle effects in these subgroups.

Of the 234 patients without a dialysis history, 66 (28.2%) were not administered IV furosemide until after ED disposition. Another 15.8% of those receiving IV furosemide were administered 10–20 mg, below the minimum dose of 40 mg recommended in current American College of Cardiology (ACC) and American Heart Association (AHA) guidelines [1, 2]. While multiple factors likely affect decisions to withhold or underdose IV furosemide, in a previous survey of ED physicians nearly 20% stated that they routinely withhold IV furosemide in ADHF patients without cardiogenic shock or baseline SBP < 90 mmHg due to a fear of hypotension [6]. Our results suggest these fears can be assuaged in patients without low baseline blood pressure.

The risk of furosemide‐related hypotension has been previously reported in chronic heart failure patients; and generally suggests more frequent occurrence of furosemide‐related hypotension in this population than we observed here in ADHF [19, 20, 21]. It makes intuitive sense that we observed hypotensive risk as far less common in ADHF patients. Acute volume overload and/or uncontrolled hypertension, common in ADHF, likely protects against hypotension compared to compensated heart failure patients.

A strength of our study design is more precise measurement of BP than standard brachial cuffs, with a monitor validated for high correlation to invasive monitoring [11, 22, 23]. Brachial BP cuffs are, by contrast, highly variable and moderately inaccurate compared to invasive BP [24], and may introduce systematic biases by sex and height [25]. Continuous monitoring is another methodological strength, allowing hypotensive episodes to be observed directly before and after treatment with precise timing and attribution of treatment effects. Standard of care BP measurements are infrequent—every 30 min to 4 h in a typical acute care setting—which makes estimation of SBP changes related to a drug like IV furosemide difficult without continuous monitoring (i.e., given a 1–2 peak effect and 6‐h duration of action). These methodological strengths are likely as rigorous as possible for a direct study of real‐world IV furosemide use, since a randomized clinical trial evaluating IV furosemide versus no furosemide for hypotension risk is unlikely to ever occur.

### 4.1 Limitations

Our results are limited by being observational rather than experimental, but an IV furosemide versus placebo trial is unlikely to occur. We used precise timing of interventions versus continuous monitoring alongside a robust multivariable‐adjustment strategy to control for selection biases and confounders. This yielded models which explained the majority of variance in SBP and hypotension overall. Nevertheless, no observational study can infer causality like a randomized trial.

Other potential limitations may apply. First, we excluded 4 patients due to missing monitoring or baseline data and observations where internal device signals indicated suboptimal signal quality, which could bias results. However, a sensitivity analysis using multiple imputation to include these patients yielded similar results. Second, the sample included few IV furosemide doses above 80–100 mg, meaning that estimates at higher doses should be treated with caution. Third, while the monitoring device used has been validated to have nearly perfect (*R* ^2^ = 0.96 [11]) correlation with invasive monitoring, it is not the gold standard. A study with strictly intra‐arterial SBP may not be feasible with as many patients as we studied, since invasive arterial monitoring is not standard or common in this population. Fourth, our study population was restricted to relatively uncomplicated ADHF episodes almost exclusively in patients with Stage C heart failure. While this patient profile represents the majority of the patients hospitalized for ADHF annually, our results do not likely apply to ADHF patients presenting with advanced (i.e., Stage D) heart failure, cardiogenic shock, or ST‐elevation myocardial infarction. Fifth, NYHA classifications prior to acute decompensation and ED arrival were unknown for patients in the cohort, and thus we could not adjust for this factor, which may have been relevant. However, this does reflect the clinical reality at these hospitals; most patients arrive at the ED without a known recent NYHA classification (e.g., due to lack of recent outpatient cardiology follow‐up or incomplete documentation) and ED providers must nevertheless make treatment decisions in the absence of this information. Sixth, while this was a preplanned analysis with a convenience sample, it is possible a larger sample could have been more robust to detect hypotensive effects (e.g., particularly in detecting interactions between co‐administrations, or acquiring more precision in point estimates of effect of IV furosemide on SBP and hypotension). Finally, we studied the association of initial IV furosemide dosing in the ED setting but not additional dosing during hospitalization. Thus, we did not evaluate effects like dose‐stacking or theoretically worsening hypotensive risk near the end of hospitalization as patients approach euvolemia.

## 5 Conclusion

In this prospective cohort study, we found that the risk of hypotension associated with IV furosemide during ADHF treatment is low and predominantly occurs at low baseline blood pressures and higher furosemide doses. These results should reassure clinicians that administration of guideline‐directed IV loop diuretic doses is hemodynamically safe in the vast majority of ADHF patients.

## Conflicts of Interest

The authors of this manuscript are disclosing the following competing interests in accordance with the ICJME guidelines: PL—Consultant: Apex Innovations, AstraZeneca, BMS, Mespere, Novartis, Cardionomics, Baim Institute, Ortho Clinical Diagnostics, Roche Diagnostics, Siemens, Hospital Quality Foundation. Research Support: American Heart Association, Beckman Coulter, Agency for Healthcare Research and Quality (AHRQ), Blue Cross Blue Shield of Michigan Foundation (BCBSMF), Emergency Medicine Foundation, Edwards Lifesciences, CardioSounds, Michigan Department of Health and Human Services, Michigan Health Endowment Fund, Patient Centered Outcome Research Institute, National Institutes of Health (NIH)/National Institute on Minority Health and Health Disparities, NIH/National Heart Lung Blood Institute (NHLBI). Other: Past Chair of the Accreditation Oversight Committee for the American College of Cardiology (ACC) and a member of the ACC's National Cardiovascular Data Registry Oversight Committee. Salary support: Edwards Lifesciences Corporation. PP—Research Support: Roche, Beckman‐Coulter, Siemens, Ortho‐Diagnostics, Abbott, and AHRQ. NEH—Research Support: BCBSMF, Indiana Clinical Translational Science Institute (CTSI). MB, KW, AD, NM, and RE report no conflicts of interest. No patents, products in development, or marketed products exist to declare in association with this research. We commit to policy adherence for sharing data and materials.

## Data Availability

The data that support the findings of this study are available on request from the corresponding author. The data are not publicly available due to privacy or ethical restrictions.
